# Cheese Analogues, an Alternative to Dietary Restrictions and Choices: The Current Scenario and Future

**DOI:** 10.3390/foods14142522

**Published:** 2025-07-18

**Authors:** Ingrid Leal, Paulo Correia, Marina Lima, Bruna Machado, Carolina de Souza

**Affiliations:** 1 National Service of Industrial Learning, SENAI Institute of Technology (IST) in Food and Beverage, SENAI CIMATEC University, Salvador 41650-010, BA, Brazil; paulo.romano85@hotmail.com (P.C.); marylima12121@gmail.com (M.L.); 2 Post-Graduate Program in Food Science, Faculty of Pharmacy, Federal University of Bahia, Campus Ondina, Salvador 40110-909, BA, Brazil; 3 National Service of Industrial Learning, SENAI Institute of Innovation (ISI) in Advanced Health Systems (CIMATEC ISI SAS), SENAI CIMATEC University, Salvador 41650-010, BA, Brazil; brunam@fieb.org.br; 4 Bromatological Analysis Department, College of Pharmacy, Federal University of Bahia, Campus Ondina, Salvador 40110-909, BA, Brazil

**Keywords:** plant-based cheese, review, technological prospect

## Abstract

The increasing demand for plant-based cheese alternatives reflects a shift toward healthier and more sustainable food choices. This study aimed to map technological trends, formulation strategies, and major challenges in the development of plant-based cheese analogues through a systematic review of the scientific literature and patents. Following the PRISMA protocol, searches were conducted in ScienceDirect and Lens.org between December 2024 and January 2025 using keywords related to cheese analogues. A total of 1553 scientific articles and 155 patents were initially retrieved. After applying inclusion and exclusion criteria, 88 articles and 66 patents were selected for detailed analysis. The results show a growing interest in this field since 2020, peaking in 2024. Data from 2025 may be limited due to the search period. Keywords were clustered into three main areas: (1) Formulation and Composition, (2) Texture and Processing, and (3) Food Safety and Consumer Acceptance. The United States leads in patent registrations (59). Valio Company and Cargill were the most active assignees, with nine and eight patents, respectively. This study highlights the importance of integrating food science and technology to improve the quality, sensory attributes, and market competitiveness of plant-based cheese analogues compared to traditional dairy products.

## 1. Introduction

Cheese analogues (CAs) are cheese-like products that result from the partial or complete substitution of components, such as milk, milk fat, or milk protein, incorporating vegetable-based substances as well as additives, such as emulsifying salts, hydrocolloids, preservatives, acidifying agents, and sometimes flavoring agents [1]. Depending on the matrix and the ingredients used in the formulation, the cheese analogues can be classified as dairy (are formulated using dairy components such as casein, caseinates, and milk fat), partial dairy (by partially substituting dairy elements, offer a blend of traditional and alternative ingredients), and non-dairy or synthetic (formulated entirely from plant-based ingredients, excluding any dairy components) [2,3]. The recent interest in vegan products and healthy alternatives has fueled the development of non-dairy CAs.

The production of alternative foods based on plant-based ingredients has been an area of growing interest and research, driven by concerns about sustainability, health, and ethics [4,5,6]. Analogue foods represent a dynamic and innovative response to current challenges, where concerns about the environmental impact of food production are prominent, as well as the pursuit of options that promote animal welfare and a balanced diet or that attend to health restrictions [7,8,9]. Such dietary transitions directly support Goal 12 of the United Nations Sustainable Development Agenda [10], which aims to “ensure sustainable consumption and production patterns,” in addition to meeting the needs of individuals with dietary restrictions, whether they are allergies, intolerances, and/or the elective exclusion of animal products [11]. From a nutritional standpoint, non-dairy CAs may offer benefits such as reduced saturated fat and cholesterol content, the absence of lactose, and the inclusion of added fibers or plant-derived micronutrients [12]. However, they often lack key nutrients naturally present in dairy cheese, such as vitamin B12, calcium in bioavailable forms, and complete proteins [12]. In a study conducted by Clegg et al. [13], which evaluated 299 commercial products, plant-based cheeses were found to contain lower levels of protein, vitamin B12, calcium, and iodine. Therefore, the widespread replacement of dairy products with plant-based alternatives may lead to reduced intake of critical nutrients over the lifespan, particularly in children and the elderly. In an evaluation of cheeses available on the Swedish market, plant-based cheeses showed lower cholesterol and saturated fat content (except for versions formulated with coconut oil), as well as higher fiber content compared to traditional dairy cheeses [12]. Similarly, in Spain, samples made from cashew nuts and tofu demonstrated a more favorable nutritional profile and potential health benefits when used as substitutes for dairy cheese [14].

Cheese, as a fundamental food in the human diet [15,16], presents unique challenges regarding its sensory and nutritional characteristics, which are conferred by the chemical composition of its primary raw material, animal-derived milk [16,17]. In this context, non-dairy cheese analogues or plant-based cheese analogues have emerged as a promising category of products [3,18,19]. Cheese analogues are products that seek to mimic the organoleptic and functional properties of conventional cheeses, but without the use of animal-derived ingredients. The evolution of this field has been marked by significant technological advances, exploring fermentation processes, texturization techniques, and the use of innovative ingredients [20,21,22]. These approaches are revolutionizing the food industry, providing alternatives not only for conscious consumers but also for those with dietary restrictions or diverse preferences [3,18,23].

Although cheese analogues may seem like a recent trend, their roots date back to ancient civilizations, where fermentation methods were used to produce cheese-like foods. Over a thousand years ago, Asian culinary cultures, such as Chinese and Japanese, were already developing soy-based products that resembled cheese [24,25]. Among other examples of traditional products made from fermented plant materials with sensory attributes similar to cheese, Ogiri from West Africa and Taioro from the Polynesian region stand out [26]. Ogiri is typical of Igbo and Yoruba cuisines (Nigeria, Ghana, Benin) and is produced through the fermentation of sesame seeds, African locust beans, or melon seeds, resulting in a pungent, strongly fermented paste with a soft texture, widely used as a condiment in traditional dishes. Taioro, traditional in Fiji, Tonga, and other Pacific islands, is made from grated coconut fermented with seawater and, in some cases, seafood, yielding a creamy-textured paste with sour and fermented flavor notes [27].

Currently, the global market for plant-based analogue products has experienced exponential growth [7,28,29] and, according to market research data conducted in 2022 by Grand View Research, it is estimated that the global cheese analogue market will reach a value of approximately USD 4.5 billion by the end of 2027, with a compound annual growth rate (CAGR) of 8.7% from 2020 to 2027 [30].

The search for information in both scientific articles and patents plays a fundamental role in the advancement of these technologies. The academic literature provides valuable insights into formulations, production processes, sensory and nutritional characteristics, and comparative evaluations with traditional products. On the other hand, the examination of patents offers a comprehensive view of technological innovations and the intellectual property strategies adopted by leading companies in this constantly evolving sector [31,32]. In this context, the present work aims to explore the recent trends and developments in the production of cheese analogues by analyzing scientific publications and patents. The discussion is structured around four thematic pillars: formulation strategies and key ingredients; technological processes for texture and flavor development; nutritional challenges and opportunities; and innovation trends and patent landscape.

## 2. Materials and Methods

This study followed the PRISMA protocol (Preferred Reporting Items for Systematic Reviews and Meta-Analyses) to ensure transparency and methodological rigor. Two different bibliographic databases were used to search articles, Science Direct and the Lens.org platform, which was used for patent searches. Platforms such as Science Direct and Lens.org are essential for technological prospecting, enabling the systematic mapping of trends, innovations, and key industry players. ScienceDirect was chosen for its high-quality peer-reviewed publications, while Lens.org was selected due to its extensive coverage of global patent data. While Science Direct provides access to high-impact scientific publications and bibliometric analyses, Lens.org integrates research articles and patents, facilitating the correlation between academic research and industrial innovation. The combined use of these tools offers a comprehensive perspective on the state of the art and technological advancements, supporting data-driven strategic decision-making. The research was conducted between December and January 2024/2025. Only documents written in English were considered, with searches in abstracts. The keywords used were (“cheese” AND (“analogue” OR “analog” OR “analog*” OR “substitute”)); the code “A23C20/00” was added for patent research, which describes “cheese; cheese preparations; making thereof (cheese substitutes A23C20/00)”. The inclusion criteria encompassed only original research articles, excluding reviews, conference papers, early access articles, book chapters, and duplicate articles.

The literature search was conducted using the Science Direct database, initially identifying 1553 records. A preliminary screening was performed to exclude review articles and publications within the 2010–2025 timeframe, resulting in the removal of 690 articles.

Following this step, 695 articles were subjected to further screening. Additional exclusion criteria were applied, eliminating studies that did not address cheese or focused exclusively on milk-based cheese, leading to the exclusion of 607 articles. After completing the selection process, a total of 88 articles were deemed eligible for analysis.

**Figure 1:**
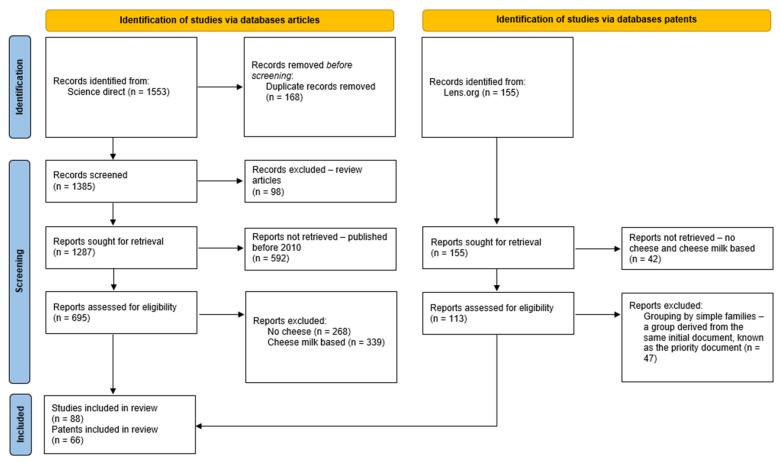
Systematic review flowchart, following the PRISMA protocol.

The selected articles were independently evaluated by two reviewers for compliance with the established criteria. Disagreements between reviewers during the screening process were resolved through discussion. When consensus could not be reached, a third independent reviewer was consulted. Figure 1 illustrates the workflow used for article selection, as recommended by the PRISMA protocol, highlighting each step of the process from the initial search to the final selection of eligible articles.

The search for patent documents was conducted using the Lens.org database, initially identifying 155 records. A preliminary screening was performed to exclude patents that did not involve cheese or were related exclusively to milk-based cheese, leading to the removal of 42 records. Following this step, 113 patent documents were subjected to further screening. At this stage, the results were grouped by simple families, which include patent documents derived from the same initial document, known as the priority document. After applying these criteria, a total of 66 eligible patent documents were selected for analysis. This methodological process ensures that only the most relevant and high-quality studies are included, providing a solid basis for the study’s conclusions.

Keyword co-occurrence analysis was performed using VOSviewer, version 1.6.20, to cluster the terms extracted from the selected articles.

## 3. Results and Discussion

**Figure 2:**
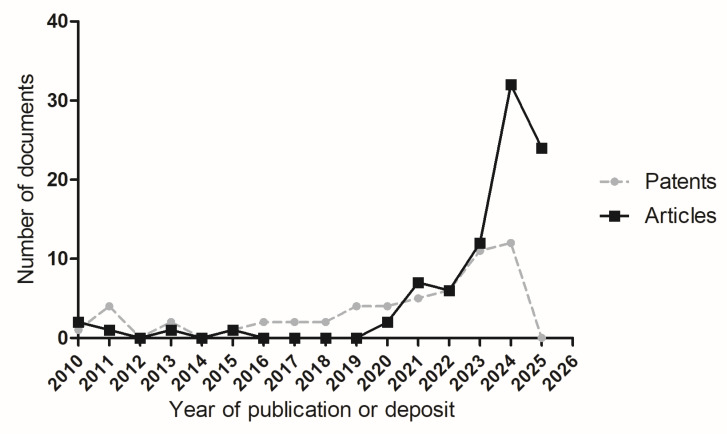
Total number of publications (articles and patents) identified in the databases evaluated over the years between 2010 and 2025.

The market for plant-based products and dairy substitutes has experienced exponential growth in recent decades [33,34]. This trend reflects a significant shift in consumption patterns, as consumers seek healthier and more sustainable alternatives to cheese products [33]. Moreover, there is a growing number of reported cases of food intolerances and allergies [35]. Figure 2 shows a trend in the evolution of article publications and patent applications over the years.

The analysis of the temporal evolution of scientific articles and patents between 2010 and 2025 (data available until January 2025) reveals distinct trends in publication and intellectual property deposition. From 2010 to 2019, the number of published articles remained relatively low, with values fluctuating between zero and two per year. A noticeable increase started in 2020, with a peak in 2024, where 32 articles were published, followed by 24 articles in 2025.

Although no patents from earlier years were excluded from the graphical dataset, the search in the Lens.org database retrieved only 10 patents between 1977 and 2009. Given the limited number of patents in this period, it was deemed more appropriate to present the same timeframe as the articles (2010–2025) in Figure 2. This ensures a more consistent and comparative visualization of the trends in scientific publications and patent filings over the years, allowing for a clearer interpretation of the recent evolution in both domains. In contrast, patent filings exhibited a different pattern. Between 2010 and 2019, there was a moderate number of patent deposits per year, ranging from one to four patents annually. A slight increase was observed between 2020 and 2023, reaching a maximum of 12 patents in 2024. However, in 2025 (until January), no patent deposits were observed. The graph clearly illustrates these trends, with a substantial rise in article publications from 2020 onwards, particularly between 2023 and 2024.

The observed increase in both scientific publications and patent filings related to plant-based cheese alternatives can be attributed to several converging factors that have shaped the food industry and consumer preferences over the past decades. One of the primary drivers is the rise in food allergies and lactose intolerance. The growing prevalence of dairy allergies, particularly to casein [36], and the increasing number of individuals diagnosed with lactose intolerance [37,38,39] have created a demand for dairy-free cheese alternatives. This demand has encouraged both academic research and technological innovations to develop formulations free of animal ingredients that replicate the sensory and functional properties of traditional cheese [40,41].

Another significant factor is the growth of veganism and plant-based diets. Ethical concerns regarding animal welfare, environmental sustainability, and health consciousness have led to a surge in consumers adopting vegan and flexitarian lifestyles [42,43]. As a result, there is increasing interest in dairy-free alternatives that offer similar texture, flavor, and nutritional value as conventional cheese, driving scientific research and patent activity in this field [44].

Additionally, challenges in dairy production and supply chain disruptions have further fueled the development of plant-based cheese substitutes [45,46,47]. Fluctuations in dairy availability, caused by climate change, increasing production costs, and geopolitical factors, have encouraged the exploration of alternative raw materials [48]. Plant-based ingredients such as nuts, legumes, and oils have gained prominence as sustainable and scalable options, stimulating research in food science and technology [49].

Regulatory changes have also played a crucial role in this evolution. The development of food regulations and labeling standards regarding plant-based dairy alternatives has expanded the market for these products [50]. Many countries have introduced new guidelines to ensure that plant-based cheeses meet safety, nutritional, and labeling requirements [51,52]. These regulations have incentivized investment in research, resulting in an increase in both scientific publications and patent registrations.

General gaps remain in legislation regarding the definition and commercialization of plant-based analogue products. Given this scenario, for example, implementing specific regulations for plant-based products in Brazil is essential to ensure transparency and consumer protection. The recent MAPA (Ministério de Agricultura, Pecuária e Abastecimento—Ministry of Agriculture, Livestock and Supply) Ordinance No. 831/2023 [53] proposes minimum identity and quality requirements for these products, but challenges remain. Establishing clear guidelines aligned with international practices can facilitate the standardization of nomenclature and prevent potential regulatory conflicts in the future. Moreover, proper regulation can support the sustainable growth of the sector, encouraging innovation and providing greater predictability for industries developing plant-based alternatives.

Lastly, technological advancements in food science have quantitatively enhanced the sensory and functional performance of plant-based cheese analogues. For example, oleogelation using phytosterol-based oleogels reduced the saturated fat content from 45.7% to just 5.2% while substantially improving hardness and meltability compared to canola oil oleogels with carnauba wax in vegan cheeses [54]. Moreover, the application of 0.25% microbial transglutaminase in cashew-based vegan cheeses increased the meltability index to 3.02 ± 0.14 and yielded an acceptable hardness of 3479 ± 15 g, significantly higher than the enzyme-free control [55]. Studies with pea protein gels demonstrated that, under high-moisture extrusion, the addition of transglutaminase significantly increased the G′ modulus (a measure of elasticity), thereby enhancing matrix firmness. Additionally, the replacement of native starches with modified variants (such as oxidized starch) improved elasticity and melting performance in patents describing processing temperatures between 62 and 85 °C [40]. Compared to earlier formulations—which relied solely on blends of starch and saturated and hydrogenated vegetable fats—these technologies offer better structure–function relationships, bringing plant-based cheese analogues closer to the sensory performance of dairy cheese. These findings support the observed increase in scientific publications and patents in the sector, reflecting tangible progress in replicating the functional and sensory behavior of conventional cheese.

These combined factors explain the noticeable increase in research publications starting in 2020 and the peak in patent filings observed in 2024. As consumer demand for plant-based cheese alternatives continues to grow, further technological advancements and regulatory adaptations are expected to shape future developments in this domain.

**Figure 3:**
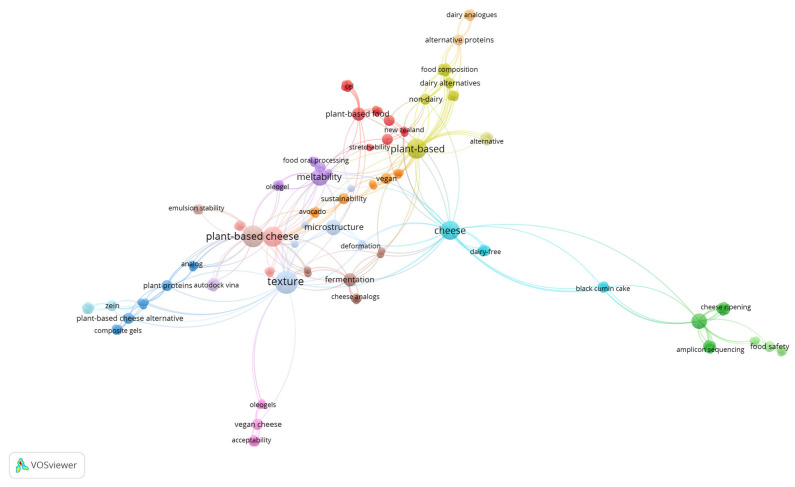
Co-occurrence map of keywords.

Among the current protection requests, there are innovations related to different raw materials and detailed descriptions of the production process. For example, Henricus et al. [56] developed a potato-based cheese analogue and its method of manufacture, consisting of a mixture of dry matter, water, potato tuber cell wall material and intercellular substances, and potato starch. The formulation excludes hydrolyzed starch, carrot, egg yolk, flour, casein, or their combinations. The production method involves providing cooked potato tuber material, refining it under high shear, cooling to form a mass, and solidifying it to obtain the cheese analogue. This cheese analogue can be used in various dishes such as pizza, lasagna, gratins, and others [56]. Figure 3 presents a co-occurrence map of keywords extracted from the analyzed articles, organized into distinct color-coded clusters. This type of analysis enables the identification of research trends, key topics, and connections between concepts related to plant-based cheese. The distribution of terms reveals the interdisciplinary nature of this field, covering aspects ranging from formulation and composition to processing and food safety.

The light blue cluster group keywords are associated with “cheese” and its alternatives, such as “dairy-free,” highlighting the relationship with lactose-free cheeses and alternative ingredients. Additionally, its connection to “cheese ripening” and “food safety” suggests a focus on the maturation and microbiological safety of plant-based cheeses, aspects that are essential for their commercial acceptance. The green cluster, in turn, emphasizes food safety and the microbiota of fermented cheeses, featuring terms such as “cheese ripening,” “amplicon sequencing,” and “food safety,” indicating the use of advanced technologies to monitor the quality and safety of these products.

The yellow cluster highlights terms related to the formulation and composition of plant-based cheeses, including “plant-based,” “dairy alternatives,” “alternative proteins,” and “food composition,” reflecting the search for alternative protein sources to improve the functionality of these products. The red cluster gathers keywords indicating challenges in replicating the properties of conventional cheeses, such as “plant-based cheese,” “stretchability,” “non-dairy,” and “sustainability,” emphasizing the importance of texture, elasticity, and sustainability in plant-based cheese production.

The purple cluster includes terms such as “food oral processing,” “meltability,” “microstructure,” and “fermentation,” highlighting the importance of processing techniques and fermentation in improving the structure and flavor of plant-based cheeses. The dark blue cluster, on the other hand, features terms like “zein,” “plant proteins autodock vina,” and “composite gels,” indicating a focus on plant protein engineering and its interactions within the food matrix. Finally, the pink cluster covers aspects of sensory acceptance, with terms such as “vegan cheese,” “acceptability,” and “oleogels,” suggesting strategies to enhance consumer perception of these products.

The organization of keywords into clusters indicates that research on plant-based cheeses is structured into three major thematic areas: (1) Formulation and Composition, focused on optimizing alternative proteins, structured fats (*oleogels*), and emulsifiers; (2) Texture and Processing, involving technologies to improve functionality, meltability, and elasticity; and (3) Food Safety and Consumer Acceptance, addressing studies on fermentation, maturation, and sensory perception. These findings reflect the current challenges and trends in the development of plant-based cheeses, emphasizing the need for integration between food science, processing technology, and microbiology to create innovative and widely accepted alternatives in the market.

Various studies have demonstrated the benefits of a plant-based diet, including the reduction in the risk of chronic diseases such as cardiovascular diseases, type 2 diabetes, and certain types of cancer [57,58,59,60]. Additionally, dairy substitutes, such as plant-based milks and vegan cheeses, often have favorable nutritional profiles, with lower saturated fat and cholesterol content, and they can be enriched with essential vitamins and minerals [61,62,63]. These products not only meet the needs of consumers with lactose intolerance or milk allergies but also provide nutritious options for a broader population.

Environmental and ethical motivations are also fundamental to the growth of the sector. The production of plant-based foods requires significantly fewer natural resources, such as water and land, and generates fewer greenhouse gas emissions compared to traditional livestock farming, especially when compared to ruminants [64]. Innovative technologies, such as precision fermentation and cellular agriculture, 3D printing, and others, are emerging as promising solutions for the production of high-quality dairy substitutes, with taste and texture similar to traditional products but with a much lower environmental and ethical impact [65,66]. These innovations are paving the way for a more sustainable and compassionate food future.

The study by Sharma et al. [65] investigated the development of a functional fermented plant-based cheese made from peanuts using *Lactobacillus rhamnosus NCDC18*. The extraction of peanut milk was optimized to a 1:6 (peanut/water) ratio, and protein coagulation was most efficient with 0.5% magnesium chloride (MgCl_2_), resulting in high solid (51.7% ± 0.04) and protein recovery (69.2% ± 0.01). Fermentation was conducted at 37 °C for 24 h, leading to significant physicochemical changes, including a pH reduction from 5.9 to 4.26 and an increase in titratable acidity (0.88% to 0.99%). Additionally, carbohydrate content decreased (from 7.72% to 5.34%), as did protein levels (from 23.38% to 20.25%) due to probiotic metabolism, resulting in proteolytic activity of 61 μg/mg. During refrigerated storage at 4 °C for 15 days, cell viability declined from 2.34 × 10^10^ to 2.78 × 10^6^ CFU/mL, highlighting the need for strategies to improve probiotic stability. The study concludes that fermented plant-based cheese represents a promising alternative to traditional dairy products, offering functional and nutritional potential, although challenges related to maintaining probiotic viability during storage remain to be addressed.

Shahbazi, Jäger, and Ettelaie [21] investigated the use of Pickering emulsions in the 3D printing of reduced-fat cheese analogues, aiming to enhance texture, thermal stability, and sensory perception. The partial replacement of oil with acetylated microcrystalline cellulose (AMCC) resulted in a more porous and homogeneous network, increased freeze resistance, and improved oral lubrication, providing greater creaminess and mouth-coating sensation. The research involved formulating emulsions with sodium caseinate, canola oil, and AMCC, followed by layer-by-layer 3D extrusion printing and structural and sensory analyses. The results indicated that AMCC improves the stability and functionality of 3D-printed plant-based cheeses, highlighting the potential of Pickering emulsions for nutritional personalization and innovation in alternative dairy products. Among the tested formulations, the one containing the highest level of AMCC (4.2%) and the lowest oil content (4%) demonstrated the most satisfactory results. This formulation exhibited enhanced 3D printability, with smoother and more stable structures, improved mechanical strength, reduced surface roughness, and greater porosity. It also showed superior freeze–thaw stability and better sensory performance, particularly in terms of creaminess, mouth-coating, and lubrication. These findings suggest that higher AMCC concentrations can effectively compensate for fat reduction while maintaining desirable structural and sensory properties in 3D-printed cheese analogues, highlighting the potential of Pickering emulsions for nutritional personalization and innovation in alternative dairy products.

Plant-based cheeses or analogue cheeses face significant sensory challenges compared to traditional cheeses, as plant proteins do not fully replicate the structural, textural, and aromatic properties of conventional dairy products [34,67]. Studies indicate that consumer acceptance is reduced due to characteristics, such as grainy texture, low melting capacity, and the presence of undesirable residual flavors, particularly those associated with plant-based sources, like soy and pea [67]. Reformulation strategies have been explored to enhance these attributes, including modified fermentation [68], in which cultures, such as *Lactobacillus*, contribute to the development of more complex and desirable sensory notes, as well as the combination of plant-based milks—such as soy, coconut, and cashew—to improve texture and mitigate herbal flavors [69,70].

Technological methods, such as thermal blanching and the use of sodium bicarbonate, have also been employed to reduce the perception of “beany flavor” and minimize the graininess of formulations [67]. Sensory analyses indicate that consumers show greater acceptance of fermented and aged plant-based cheeses, which exhibit enhanced aromatic complexity and textural improvements. However, despite advancements, there remains a perceptible distinction between analogue and conventional cheeses, suggesting the need for innovative formulation and processing strategies to achieve broader acceptance [34]. Therefore, research in this field should focus on integrating advanced sensory methodologies and market segmentation, enabling the development of products that meet both organoleptic expectations and nutritional and environmental demands [71].

**Figure 4:**
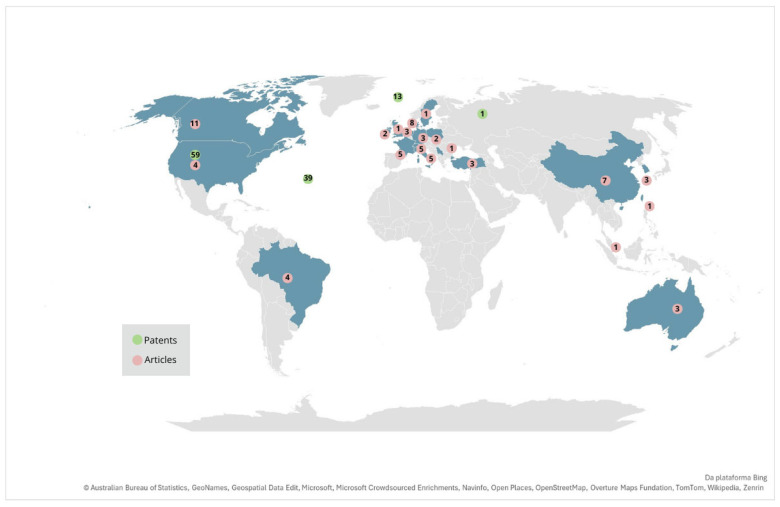
Countries of patent deposit (green) and origin of the main authors of the articles (red).

Understanding the locations of patent filings and the affiliations of academic article authors is essential for elucidating technologies, as these elements provide insights into legal protection, commercial strategy, and the credibility of innovations. The location of a patent filing reveals where the technology is legally protected, indicating target markets and the origin of the innovation, while author affiliations show the provenance of the research, institutional collaboration, and the funding involved [72]. Together, these factors help us to better understand the development, relevance, and impact of technologies. Figure 4 shows the distribution across the countries of patents and articles selected in the present work.

Figure 4 presents a global map illustrating the distribution of patents (green) and scientific articles (pink) related to plant-based cheese. The United States leads in technological innovation, with 59 registered patents and four articles, reflecting strong investment from major food companies and startups focused on protecting their technologies. A recent report published by “USA FACTS” states that “despite increased production, dairy consumption is declining: Americans drink 47% less milk today than in 1975,” and part of this can be explained by the consumption of plant-based products as substitutes for dairy [73,74]. The plant-based cheese market in the United States has experienced significant growth in recent years. In 2021, retail sales of plant-based foods in the U.S. increased by 6.2% compared to 2020, reaching USD 7.4 billion, with the plant-based cheese segment growing by 7%, while conventional cheese sales declined by 2% over the same period. It is estimated that over 30 million people in the U.S. are lactose intolerant, contributing to the growing demand for non-dairy alternatives, such as vegan cheese. On a global scale, the vegan cheese market was valued at approximately USD 2 billion in 2022 and is expected to reach USD 4.5 billion by 2030, with a CAGR of 10.5% over this period [75,76]. These figures reflect a strong and growing demand for plant-based dairy alternatives, highlighting a shift toward more sustainable food choices [19].

Additionally, 39 patents were deposited through WIPO and 13 through the European Patent Office, highlighting the use of international patent systems. Depositing patents via the Patent Cooperation Treaty (PCT) offers several advantages, including international protection with a single application, delayed national filing costs, extended time for market assessment, streamlined examination processes, and increased licensing opportunities. This system allows applicants to secure priority in multiple jurisdictions, reducing initial expenses while evaluating commercial potential before committing to full national filings. In contrast, Europe and Asia emerge as key academic research centers, with countries such as Germany, France, and Italy contributing significantly to scientific publications on plant-based cheese formulation and improvement. In Asia, China leads with seven published articles, indicating growing interest in plant-based product innovation, though patent activity in these regions remains relatively low, suggesting research is still in an exploratory phase or that intellectual property protection strategies differ from those in the U.S.

In Latin America, Brazil has only four scientific articles and no significant patents, indicating an early stage of research and development in this field, possibly due to technological barriers, limited innovation incentives, or challenges in translating academic research into commercial products. The map highlights a discrepancy between scientific production and patent protection, showing that while some countries excel in academic research, others focus on securing intellectual property. These findings reinforce the need for greater investment in research, development, and innovation to strengthen the global plant-based cheese market and expand technological advancements, particularly in regions where this sector is still emerging.

Many countries have been suffering from the impacts of climate change, which are directly related to issues of food insecurity [77,78]. Interest in dairy substitutes in different regions may also be related to health risks, given the use of pesticides and antibiotics in lactating cows [79]. Furthermore, with the increasing population, it is necessary to consider the use of new food sources to ensure a supply of sufficient quality and quantity. Thus, studies and patents support the need for dairy alternatives, promoting sustainable practices and the optimized use of resources to mitigate negative impacts and support regional economies, promoting sustainable practices and the optimized use of resources to mitigate negative impacts and strengthen local agri-food systems, small-scale production, and regional socio-economic development. The data presented in Figure 4 show that research on “cheese” and “analogue” is globally distributed but particularly concentrated in countries with strong traditions in food science and technology. This distribution reflects the growing importance of cheese analogues as a viable and innovative alternative to traditional dairy products, driven by factors such as health, sustainability, and food innovation.

**Figure 5:**
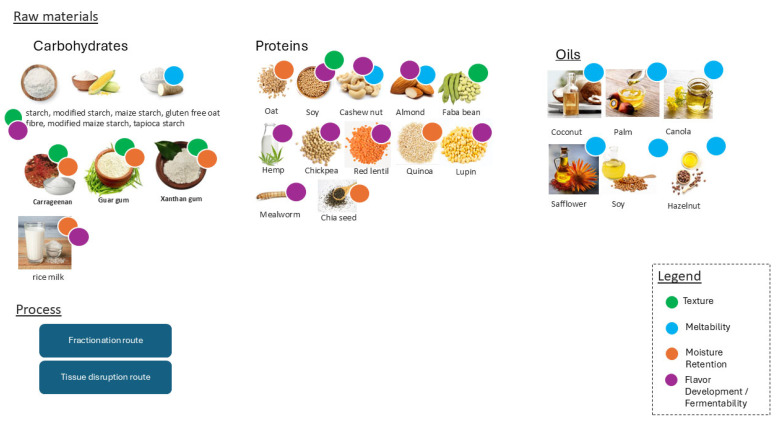
Functional roles of raw materials used in plant-based cheese analogues. Ingredients are annotated according to their primary technological contributions: texture enhancement (green), meltability (blue), moisture retention (orange), and flavor development/fermentability (violet). Classification based on claims and usage in selected scientific publications and patents.

The main properties and compositions of the ingredients used in plant-based cheese analogues are summarized in Figure 5, categorizing them based on their functional groups: carbohydrates, proteins, and fats. Each of these ingredient categories plays a crucial role in replicating the texture, meltability, flavor, and overall sensory experience of conventional cheese. Polysaccharides play a crucial role in the formulation of plant-based cheese analogues by contributing to texture, structure, and moisture retention. Starches, particularly from sources like tapioca, potato, and corn, are widely used due to their ability to gelatinize upon heating and retrograde upon cooling, forming a viscoelastic network that mimics the firmness and elasticity of dairy cheese [80,81]. This network helps to trap water, fats, and proteins, enhancing the overall consistency and meltability of the product. Additionally, carbohydrates can act as stabilizers and thickeners, improving mouthfeel and preventing syneresis (water release) during storage. Its functional properties are essential to mimic sensory attributes [82,83,84].

Proteins are fundamental in the development of plant-based cheese analogues due to their multiple functional roles [85,86]. They contribute to structure formation by creating a protein network that provides firmness and meltability, primarily through water and fat retention [85,87,88]. Their emulsifying capacity enables the stable dispersion of fat within the matrix, essential for uniform texture and mouthfeel. The textural properties of the final product are influenced by protein solubility, thermal stability, and interfacial activity, which help mimic the elasticity of dairy cheese [21]. Some proteins, such as zein from corn, offer film-forming and encapsulation abilities, enhancing the stability and smoothness of the product. To further improve these functionalities, proteins can be modified through fermentation, enzymatic treatment, or crosslinking, which enhances both structural and sensory characteristics [86,89].

Lipids are fundamental in plant-based cheese formulations, influencing texture, meltability, flavor, and nutritional profile [90,91,92]. Unlike dairy fats, their plant-based counterparts display diverse melting behaviors due to variations in fatty acid composition and processing. Fats with higher saturation levels and longer chains, such as coconut oil, are more effective in mimicking the structural attributes of traditional cheese.

Fats play a fundamental role in plant-based cheese analogues, influencing texture, melting behavior, sensory properties, and nutritional composition [90,91,92]. Unlike dairy fats, which naturally provide creaminess and structure, plant-based fats vary in fatty acid composition and melting behavior, depending on their botanical origin and extraction method. Common oils such as sunflower, corn, and canola are rich in unsaturated fatty acids, making them liquid at room temperature and unsuitable for forming solid fat crystal networks, which are essential for cheese texture [91]. Historically, hydrogenation was used to solidify these oils, but concerns over trans fat formation and associated cardiovascular risks have led to its decline [93]. Instead, manufacturers blend solid fats (e.g., coconut oil, cocoa butter, and palm oil)—which are typically high in saturated fats and associated with potential health risks when consumed excessively—with liquid oils to optimize the solid fat content (SFC) versus temperature profile, ensuring firmness, meltability, and desirable sensory attributes. However, many solid fats are high in saturated fats, raising health concerns when consumed in excess [94]. Additionally, polyunsaturated fats, such as flaxseed and algal oils, offer potential health benefits but are highly susceptible to oxidation, leading to rancidity and off-flavors [95]. To improve stability and shelf life, strategies such as oxygen reduction, antioxidant incorporation, and oil-in-water structuring are used [96]. Among the various plant-derived fats used in plant-based cheese formulations—including avocado, canola, coconut, palm, and soybean oils—only a few remain solid at room temperature, making them essential for texture enhancement. Through careful blending of solid and liquid fats, manufacturers can replicate dairy cheese properties, balancing functionality, health considerations, and sustainability in plant-based cheese production.

Some articles have demonstrated the potential use of different matrices to produce cheese analogues. The study by Ferawati et al. [97] focused on evaluating the use of legume flours, specifically cooked and toasted yellow pea and fava bean flours, as raw materials to create functional and healthier alternatives in the plant-based cheese market. The research investigated the effects of different stabilizers and processing conditions on the texture and overall quality of cheese analogues. It was found that pre-treating the legumes reduced antinutrients and improved functional properties, such as water absorption capacity and gel formation. Additionally, the samples showed significantly higher crude protein and fiber contents compared to commercial vegan cheese analogues, while the fat content was lower.

The study by Esen, Guneser, and Akyuz [98] aimed to evaluate the physicochemical, microbiological, and sensory properties, along with the aromatic profile, of cheese analogues produced from plant protein sources (chickpea, lentil, and bean flours) and dairy sources. The aromatic profile analysis, conducted by gas chromatography–mass spectrometry, identified and quantified volatile compounds, differentiating plant-based cheeses from dairy cheeses. The results showed that cheeses based on plant proteins are ideal options for individuals following a plant-based diet, offering significant economic benefits and new flavors to the gastronomic market.

Although soy and its derivatives (mainly oil and flour) are the ingredients mentioned in the selected studies for evaluation, other authors investigate the potential use of rice milk, chia seed, and hazelnut oil [99]; soy and cashew nut milk [100]; soy and coconut milk [101]; faba bean and mealworm flours [102]; and emulsion gels by blending cationic and anionic potato protein-coated oil [90]. In addition to the main ingredients, studies on process aids, such as coagulants and fermentative agents, are extremely necessary to understand the production process of cheese analogues free of animal-derived ingredients (excluding microorganisms).

The production of plant-based cheeses involves different phase transitions than conventional cheese, as it requires structuring plant-derived ingredients to mimic the characteristics of dairy cheese. There are two main approaches to this production: the “Fractionation Route” and the “Tissue Disruption Route” (Figure 5).

In the “Fractionation Route”, raw plant material is processed to isolate and purify functional ingredients, such as polysaccharides, proteins, and fats. Proteins can be extracted from soy, peas, beans, lupin, and potato, while starches are isolated from corn, peas, tapioca, and potato. Other hydrocolloids are obtained from terrestrial plants (pectin, guar gum, locust bean gum, and cellulose), marine algae (agar, alginate, and carrageenan), or microorganisms (xanthan gum). Plant-based fats, which can be liquid or semi-solid at room temperature, are often derived from coconut, palm, cocoa, soybean, sunflower, or rapeseed. These ingredients are dissolved in water and combined with oil to form a plant-based emulsion, which is then solidified through various processes, including heating, cooling, pH adjustment, enzymatic, or salt addition. During these steps, proteins solubilize, denature, and aggregate; starches gelatinize; fats emulsify, melt, and crystallize; and gums undergo conformational transitions [103,104,105]. As a result, this route involves multiple phase transitions to produce the final plant-based cheese: solid (raw plant material) → liquid (extraction/fractionation) → solid (dry ingredients) → liquid (emulsion) → solid (plant-based cheese).

Conversely, the tissue disruption route utilizes whole plant raw materials for plant-based cheese production, incorporating ingredients such as soy, peas, or nuts. The plant materials are soaked for softening and then ground, forming a colloidal dispersion containing oil globules, fragmented plant tissues, dissolved biopolymers, sugars, and salts. This dispersion can be converted into plant-based cheese through a sol–gel transition, using the same methods applied in the fractionation route. However, unlike the fractionation route, the tissue disruption approach requires fewer phase transitions, making it a more direct and potentially energy-efficient process [100,106,107,108]. The key phase transitions in this method include solid (plant raw material) → liquid (emulsion) → solid (plant-based cheese).

To produce cheese analogues by the tissue disruption route, several studies have explored lactic acid fermentation as a method for producing plant-based cheese analogues, primarily using soy milk as a base. Instead of traditional coagulants, like glucono-delta-lactone or calcium, some studies have used starter cultures such as *Bifidobacterium animalis* subsp. *lactis Bb-12*, *Lactobacillus acidophilus La-5*, and *Streptococcus thermophilus* to induce coagulation [109]. The final pH influenced the structure, yielding a quark-type cheese at pH 5.7 and a firmer soy cheese at pH 4.8. Other approaches have used *Lactobacillus fermentum* and *S. thermophilus* to produce semi-hard soy-based cheese [110] and *Lactobacillus casei* to create spreadable cheeses [111]. The addition of papain has been shown to improve texture and sensory attributes by reducing protein particle size in soy cheese spreads [68]. *Lactobacillus rhamnosus* has also been used to ferment soy milk into plant cheese, with curd formation followed by pressing and salting [112]. While most studies focus on soy-based cheeses, recent research suggests that lupin and other legume-based plant milks could be promising alternatives [113,114]. Further research is needed to optimize fermentation techniques, texture, and sensory properties to support the development of a wider range of plant-based cheese styles and formulations, including alternative legumes.

The distinction between these two approaches lies in the mechanism driving the sol–gel transition. In the fractionation route, this transition is facilitated by combining fractionated and isolated plant-based ingredients from different sources. In contrast, in the tissue disruption route, the transition primarily occurs due to the intrinsic components of the original raw material, which is used either as a whole (e.g., nut paste) or with minimal separation steps (e.g., plant milk). The resulting coagulated structures can be classified based on the type of coagulation process used, which may involve self-association, enzymatic, acidic, ionic, thermal, or combined methods. The choice of route and coagulation process directly influences the texture, stability, and sensory profile of the final plant-based cheese.

As previously stated, there are different methods for the coagulation process in the formation of plant-based cheeses.

The study by Ndife, Imade, and Samaila [115] investigated and compared the effects of different coagulants (alum, *Moringa oleifera* seed extract, tamarind seed extract, and lemon juice) on the physical, chemical, and sensory properties of soy cheese. Among the tested coagulants, *Moringa oleifera* seed extract stood out, providing higher yield and acidity, as well as superior sensory quality and higher nutrient content compared to other coagulants. Soy cheese coagulated with alum salt also showed favorable organoleptic properties, although with lower nutritional values. The study concluded that the choice of coagulant is crucial for achieving desired sensory qualities, nutritional benefits, and consumer acceptance, suggesting that producing soy cheese with Moringa oleifera extract could serve as a functional food option. It is important to note that, despite its positive results, Moringa oleifera still faces restrictions on its use as a food ingredient or with pharmaceutical properties in some countries, such as Brazil [116].

The study by Xie et al. [117] investigated the impact of germinating soybean and lupin seeds and fermenting with *Bacillus* spp. on the microbiological and physicochemical characteristics of plant-based cheese analogues. The objective was to understand how these processes influence the ripening of plant-based cheeses, focusing on the control of microbial growth, flavor development, and overall enhancement of the final product’s quality. The research utilized *Bacillus velezensis* and *Bacillus amyloliquefaciens* during the fermentation process, as well as *Lactiplantibacillus plantarum* and *Lactococcus lactis* as starter cultures for acidification. Microbial counts were assessed using selective agar plating (LB, M17, MRS, VRBG), and microbial community profiling was performed through full-length 16S rRNA gene sequencing. Physicochemical analyses included pH measurements, moisture determination by gravimetric analysis, and the quantification of free amino nitrogen using a spectrophotometric assay. The combination of germination with lactic acid bacteria and *Bacillus* spp. showed potential in improving the quality of matured plant-based cheeses, highlighting positive effects on flavor development and a reduction in hygienic risks.

Table 1 illustrates the distribution of the primary patent applicants, including both companies and individual inventors, in the field of plant-based cheese and dairy alternatives. The leading applicant is Valio Ltd., with a total of nine patents, followed closely by Cargill Inc. (eight patents) and Leprino Foods Co. (seven patents). Other notable contributors include New Culture Inc. and Papadakis Peter, each with six patents, demonstrating a balance between corporate and individual innovation.

Several multinational companies, such as Nestlé SA, Arla Foods Amba, and Oatly AB, each hold multiple patent filings (four to three patents), highlighting their active role in developing alternative dairy products. Additionally, organizations such as Eat Just Inc. and Dow Global Technologies LLC are also represented, reinforcing the diverse industrial participation in plant-based cheese innovations. The presence of independent inventors, including Domazakis Emmanouil and Wolford Miles D, further indicates that individual contributions remain relevant in this sector.

Overall, these findings highlight a competitive and evolving landscape, with major food and biotechnology companies, as well as independent inventors, driving innovation in the development of plant-based cheese alternatives. The combination of corporate research and individual expertise suggests a multi-faceted approach to technological advancements, ensuring continuous progress in plant-based dairy substitutes.

Valio produces high-quality dairy and plant-based products, catering to both consumers and the wholesale sector. The company is committed to sustainability, participating in circular economic initiatives. The main patents, US 12,144,361 B2 [118], US 2022/0273001 A1 [119], and US 11,071,312 B2 [120], focus on the development of alternative protein products, emphasizing thermal stability and applications as meat or cheese substitutes. US 12,144,361 B2 describes an acidified protein product based on casein, structured in grains or compacted blocks, differing from traditional cheeses by its resistance to melting and adhesion to cookware when grilled or fried. US 2022/0273001 A1 also presents an acidified protein product but emphasizes the use of crosslinking enzymes to modify the protein structure, ensuring greater stability under heat and allowing its use as both a cheese and meat substitute. Meanwhile, US 11,071,312 B2 focuses on a thermally stable plant-based protein product, formulated from a plant protein concentrate combined with hydrocolloids and, optionally, dairy proteins, such as casein or crumbled cheese, resulting in a food product that can be shaped into various forms and prepared by frying, grilling, or microwaving without losing its structure. While the first two patents focus on dairy-based proteins and acidification processes, the third stands out for using plant-based proteins and hydrocolloids to achieve thermal stability, offering a fully plant-based or hybrid alternative to traditional meat and cheese products.

Cargill is an American multinational corporation operating in the agro-industrial, food, and financial sectors with a global presence. Founded in 1865, the company has over 150 years of experience and operates in 70 countries. Its portfolio encompasses the production and commercialization of food ingredients, grains, oils, meats, animal nutrition, and industrial products, in addition to providing financial services and solutions for the agricultural supply chain. Cargill stands out for its commitment to sustainability, environmental impact reduction, and community development, working in partnership with farmers, customers, governments, and organizations.

Four of the analyzed patents focus on innovations in the formulation and processing of cheese analogues, each targeting different aspects to optimize the final product. WO 2023/081651 A1 [121] presents a vegan cheese formulated with corn protein isolate (CPI), which enhances coloration after cooking, increases protein content, and reduces fragility while allowing flavor incorporation without requiring masking agents. The process involves hydrating CPI, combining it with oil and starch, and subjecting the mixture to controlled heating between 50 °C and 95 °C, yielding a texture closer to that of traditional cheese. In contrast, US 2019/0059408 A1 [122] focuses on optimizing texture and melting properties by replacing potato starch with modified starches from corn, tapioca, and sago, which provide greater elasticity, firmness, and melting capacity. The manufacturing method consists of blending modified starches with vegetable fat, emulsifying salts, and casein, followed by cooking at temperatures between 62 °C and 85 °C, ensuring consistency similar to traditional cheeses while reducing costs and enhancing formulation flexibility. EP 2,904,907 B1 [123], on the other hand, proposes a differentiated approach by eliminating synthetic proteins and emulsifiers, using porous starch as a stabilizer. This method enables the creation of food emulsions for cheese analogues and meat products without the need for casein or other emulsifiers. The process involves melting lipids, mixing them with dry ingredients, and adding water to form a stable emulsion, offering advantages such as a reduction in artificial ingredients and improved liquid retention. Finally, WO 2017/040577 A1 [124] aims to reduce cheese production costs without compromising essential properties by replacing part of the proteins with modified pyrodextrins derived from starch. These pyrodextrins enhance water and fat retention while maintaining the melting, elasticity, and firmness of cheese. The process includes the chemical modification of starch, followed by its incorporation into the cheese formulation, ensuring a texture comparable to traditional cheeses with reduced dependence on dairy proteins. Thus, WO 2023/081651 A1 focuses on formulating a vegan cheese with enhanced nutritional properties, US 2019/0059408 A1 optimizes texture and melting using modified starches, EP 2,904,907 B1 offers an alternative free from artificial stabilizers, and WO 2017/040577 A1 seeks cost reduction while preserving the sensory attributes of cheese.

Table 2 provides a summary of various patents related to plant-based cheese products or their respective processes. The table highlights the key inputs and methods claimed in each patent, offering insights into the innovative approaches being developed in the field of plant-based cheese production. From the use of specific vegetable fats, starches, and proteins to advanced methods for improving cheese meltability and stretchability, these patents represent significant advancements in creating high-quality, plant-based cheese alternatives suitable for diverse culinary applications. The patents listed range from specific product compositions to detailed process descriptions, reflecting the broad scope of innovation in this area.

In general, there is a consonance between the evaluated patent documents and articles when detailing the ingredient list of “cheese analogues” products (Table 2). This demonstrates that there are significant R&D efforts from ingredient companies, food processing companies and startups, and universities aimed at continuously improving the nutritional properties, sensory characteristics, and performance of the products. Furthermore, it can be observed that different techniques can be applied, such as blends for powdered, flaked, or formed products (Parmesan Flavored Grated Cheese and Lactose-Free Grated Parmesan) and fermentation for the others. The use of fats and hydrocolloids to aid in the development of the products is also noted.

As demonstrated throughout this study, plant-based cheeses represent a promising alternative to traditional dairy cheeses, offering both nutritional benefits and environmental sustainability [1,3,129,130]. However, several key aspects remain that present new opportunities for research aimed at improving the performance and mimicry of these products. For example, a deeper understanding of material science and protein chemistry will enable greater predictability in texture, meltability, and structural behavior. The development of complex viscoelastic protein networks is an emerging trend in this field [19]. Researchers are currently working on processes to improve the functionality of plant proteins, such as legume and microalgae fractions, to better replicate the rheological and sensory properties of conventional cheeses, and other researchers are looking to reduce or eliminate off-flavors commonly found in legume-based ingredients. Masiá et al. investigated the fermentation of pea protein emulsions to improve their functional and sensory properties. Blends containing Vega™ Harmony and Vega™ Classic acidified more rapidly and were more effective in reducing compounds associated with green and beany off-flavors typically found in pea-based matrices. The presence of *Lactobacillus plantarum* and *Lactobacillus casei* in the formulations was associated with a decrease in gel firmness. Notably, dairy-like volatile compounds were detected across all fermented samples, with their levels varying depending on the specific bacterial blend used [131].

Another important aspect that could contribute to advances in the sector is the diversification of protein sources, including the use of coproducts. In a recent study, Levy et al. [132] employed potato protein isolate (PPI), derived from potato juice, as a promising functional ingredient for plant-based fermented formulations. The study also applied high-pressure homogenization (HPH) to emulsions of PPI and canola oil, resulting in yogurt-like products with gel-like behavior, clear appearance, and high physical stability, even in the absence of additional stabilizers. The particle size reduction promoted by HPH facilitated the formation of fine emulsions with excellent water-holding capacity and resistance to phase separation. These findings indicate that PPI, especially when subjected to functional physical treatments, such as HPH, may be a viable alternative for use in structured products like plant-based cheeses, contributing to improved texture, stability, and sensory profile [132].

Another crucial aspect is the aging process and sensory optimization of plant-based cheeses. In traditional dairy cheese, fermentation and microbial interactions play a significant role in the development of final flavor and texture. Early studies suggest that the same microbial cultures used in dairy fermentation yield entirely different sensory profiles when applied to plant-based substrates, such as pea protein gels, highlighting the need for substrate-specific fermentation strategies. However, there remains a significant knowledge gap regarding how different starter cultures influence the sensory attributes, physicochemical properties, and overall processing of plant-based cheeses, making this a critical area for further investigation. The use of ripening-associated cultures, such as *Penicillium camemberti* and *Geotrichum candidum,* has already shown promising results in plant-based alternatives, despite differences in fatty acid composition [133].

Melting properties are another fundamental challenge in plant-based cheese development, as most commercial alternatives exhibit significantly lower meltability compared to conventional dairy cheese. This issue has been linked to the widespread use of thermo-irreversible starches as gelling agents, which prevents the desired melting behavior. Some studies indicate that zein, a maize protein, could enhance meltability due to its ability to plasticize at high temperatures, mimicking the behavior of casein networks [134,135]. Similarly, preliminary findings suggest that soy, rubisco, moong bean [136], and pea protein-xanthan mixtures may form thermoreversible gels, but the mechanisms behind these interactions require further investigation to optimize their functionality in plant-based cheese formulations.

A qualitative analysis of the selected scientific publications and patents reveals that plant-based cheese analogues differ substantially from conventional dairy cheeses in terms of raw materials, processing techniques, and nutritional characteristics (Table 3). While dairy cheeses rely on the coagulation of casein micelles from animal milk followed by enzymatic or microbial ripening processes, plant-based analogues utilize diverse protein sources—such as soy, cashew, almond, pea, and potato—combined with hydrocolloids (e.g., carrageenan, xanthan gum), modified starches, and emulsifiers to mimic texture and mouthfeel [56,67,70,99,137,138,139,140]. Fermentation, commonly employed in traditional cheeses to develop flavor complexity and improve digestibility, is still underutilized in plant-based formulations, with most products relying on added flavors or short-term fermentation [117,131,138]. Nutritionally, plant-based cheeses often have lower protein content and lack key micronutrients naturally present in dairy, such as calcium, vitamin B12, and iodine, although some formulations are fortified [12,13,40,139]. In contrast, plant-based alternatives may offer benefits such as lower saturated fat (especially in formulations without coconut oil), the absence of cholesterol, and higher dietary fiber content [12,54,99,129]. These differences underscore the need for continued research focused not only on sensory and structural replication but also on improving nutritional equivalency and consumer acceptance.

Beyond texture and sensory attributes, the nutritional profile and sustainability of plant-based cheeses must also be carefully considered. While these products are often marketed as healthier alternatives, many formulations rely on high levels of saturated fats, salts, and starches to achieve the desired texture and mouthfeel, raising concerns about their overall nutritional quality. Developing healthier formulations that incorporate unsaturated fats while maintaining desirable structural properties is a major challenge. Additionally, ensuring the sustainability of plant-based cheeses involves assessing their environmental impact throughout the production chain, from ingredient sourcing to processing and distribution. Advanced encapsulation technologies, such as nanoemulsions, may offer innovative solutions by allowing the incorporation of bioactive compounds, like omega-3 fatty acids, vitamins, and nutraceuticals, enhancing both the nutritional value and functional properties of plant-based cheese analogues.

Lastly, food safety remains an underexplored area in the development of plant-based cheeses. Unlike traditional dairy cheese, which has well-established safety protocols and regulations, plant-based alternatives require dedicated research to ensure microbiological stability, proper storage conditions, and the absence of contaminants. Given the growing consumer demand for plant-based products, it is essential to establish rigorous food safety standards that guarantee that these cheeses are not only nutritious and palatable but also safe for consumption. Addressing these interconnected challenges—structuring, aging, melting, nutrition, sustainability, and safety—will be crucial for advancing the quality and acceptance of plant-based cheeses, paving the way for a future where these products can rival traditional cheeses in both taste and functionality.

## 4. Conclusions

This review provides a dual-layered analysis of both scientific publications and patent filings, enabling a comprehensive understanding of technological trends, formulation strategies, and innovation gaps in plant-based cheese analogues. By integrating these two data sources, the study offers a perspective on the interplay between academic research and industrial innovation, identifying underexplored areas and emerging technological priorities.

The present study explored trends and innovations related to the production of plant-based cheese analogues, addressing technological advancements, sensory and structural challenges, and regulatory and sustainability issues. The analysis revealed that, despite the exponential growth of the sector and the increasing number of academic studies and patents, significant challenges remain. The structuring of plant proteins continues to be one of the major barriers. The understanding of the ingredients, molecular and colloidal interactions, and the technologies that influence the texture and functionality of cheese analogues has been improved over the years.

Additionally, the maturation and fermentation processes of these products remain underexplored, highlighting the need for the development of specific microbial cultures to enhance flavor and texture while mitigating undesirable sensory notes. The melting properties of plant-based cheeses also represent a significant challenge, as the commonly used thermoirreversible starches prevent meltability similar to that of dairy cheeses. Promising alternatives include the use of specific plant proteins, such as zein, and the formation of thermoreversible gels through innovative protein combinations.

From a nutritional and environmental perspective, there is great potential for product improvement, as many formulations still rely on high levels of saturated fats and starches to ensure texture and stability. Emerging technologies, such as nanoemulsions and bioactive encapsulation, could serve as effective strategies to enhance the nutritional profile of these cheeses by incorporating beneficial compounds such as omega-3 fatty acids and vitamins. Lastly, the food security of plant-based cheeses remains an underexplored area, emphasizing the importance of developing specific protocols to ensure microbiological stability and quality throughout storage.

Thus, the advancement of plant-based cheese analogues depends on the integration of scientific and technological knowledge to optimize their formulation, processing, and sensory acceptance. The continuous growth of the market and technical and technological knowledge, based on articles and patents, both in the development of new processes and for the ingredients sector, suggests that these products may, in the future, fully or partially replace traditional cheeses in terms of flavor, texture and functionality, meeting both the demands of sustainability and the needs of consumers with dietary restrictions.

One limitation of this study is that the search was conducted exclusively through the ScienceDirect database, which may have restricted access to relevant studies available on other platforms. Additionally, the timeframe of 2015 to 2025 was selected to focus on the most recent innovations, but this may have excluded earlier research or publications not yet indexed.

## Figures and Tables

**Table 1:** Distribution of the main patent applicants.

Empresa	Patents
Valio Ltd.	9
Cargill Inc.	8
Leprino Foods Co.	7
Papadakis Peter	6
New Culture Inc.	6
Damazakis Emmanouil	5
Wofford Miles D	4
Netle As	4
Mevgal S a Dairy Products Industry Trad	4
Creta Farms As Ind.	4
Arla Foods Amba	4
Allied Blending & Ingredient Inc.	4
Eat Just Inc.	3
Dow Global Technologies Llc.	3
Oatly Ab	3

**Table 2:** Overview of patents and main claims regarding plant-based cheese inputs and processes.

Description	Product or Process	Reference
Consisting of an oat base (2–8% dry matter), vegetable fat (20–30%), starch (20–30%), native or modified, such as potato starch, vegetable protein (1–4%), potatoes, peas, or legumes, stabilizer (0.2–1%), and water (35–45%).	Product	[41]
Methods for producing cheese analogues with improved meltability and stretchability during heating have been developed. These methods involve using enzymes such as glucose oxidase, α-glucosidase, and transglutaminase on raw cheese or mixtures containing plant-derived oil and starch. The processes aim to suppress the decrease in texture and stretchability of cheese during heating. This innovative approach applies to various cheeses, including mozzarella, Gouda, and Cheddar, and offers a way to enhance the quality and performance of cheese analogues in culinary applications.	Process	[125]
The plant cheese analogue comprises the following raw materials: a starch raw material, a protein raw material, and a grease raw material.	Product	[126]
Describes a plant-based half-hard cheese comprising 5–35% vegetable fat, 1–45% starches and/or modified starches, 1–7% lecithin (such as soy, sunflower, cottonseed, or rapeseed lecithin), with the remainder being water. It may also include 0.1–20% plant proteins, such as lentil, fava, or pea protein. The vegetable fat can be coconut fat, preferably non-hydrogenated and palm-oil-free. The preparation method involves mixing the ingredients under shear to form a homogeneous emulsion.	Product and process	[127]
Describes a soybean and nut plant cheese made using a composite leavening agent (probiotics and lactic acid bacteria).	Product	[128]
The patent protects both the formulation of the dry blend and the process for manufacturing the cheese analogue. The main focus is the partial or complete replacement of casein with starches and stabilizers, creating a more affordable, healthier, and versatile cheese. This allows for catering to vegan markets, reducing production costs, and offering a product with sensory characteristics very similar to conventional cheese.	Product and process	[105]

**Table 3:** Technological and nutritional comparison between dairy and plant-based cheeses.

Aspect	Dairy Cheese	Plant-Based Cheese Analogues	Reference
Main protein source	Casein (animal milk)	Soy, cashew, almond, pea, potato, rice proteins	[56,67,70,99,137]
Coagulation agent	Rennet; acidification	Hydrocolloids, emulsifiers, starches	[140,141,142]
Texture development	Enzymatic coagulation + aging	Texturization via hydrocolloids, starches, oils, sometimes extrusion or gelling agents	[16,22,129,131]
Flavor development	Fermentation and ripening (microbial, enzymatic)	Added flavorings; limited fermentation	[117,131,138]
Nutritional profile	High in complete protein, calcium, B12, iodine	Often lower in protein and micronutrients; may be fortified; higher fiber or protein content	[12,13,40,139]
Lipid profile	High in saturated fat and cholesterol	Lower saturated fat and no cholesterol (except with coconut oil)	[12,54,99,129]
Processing complexity	Traditional processes (milk treatment, curdling, ripening)	Requires ingredient functionalization and texture engineering	[21,143]
Cost–benefit	High yield from dairy; established supply chain	Ingredient cost varies; may be higher in clean-label or nut-based formulations	\

## Data Availability

The original contributions presented in this study are included in the article. Further inquiries can be directed to the corresponding authors.
